# Wumei extract modulates inflammation and oral microbiota for radiation-induced oral mucositis

**DOI:** 10.3389/fmicb.2026.1837933

**Published:** 2026-08-04

**Authors:** Yumeng Wu, Qizhao Ma, Yue Yu, Jingwei Cao, Jiantao Wang, Jing Zou, Yan Wang

**Affiliations:** 1 State Key Laboratory of Oral Diseases, National Center for Stomatology, National Clinical Research Center for Oral Diseases, West China Hospital of Stomatology, Sichuan University, Chengdu, China; 2 Department of Pediatric Dentistry, West China Hospital of Stomatology, Sichuan University, Chengdu, China; 3 Department of Radiation Oncology, Cancer Center, West China Hospital, Sichuan University, Chengdu, China; 4 Lung Cancer Center/Lung Cancer Institute, West China Hospital, Sichuan University, Chengdu, China

**Keywords:** epithelial regeneration, inflammation, oral microbiota, radiation-induced oral mucositis, Wumei extract

## Abstract

**Introduction:**

Radiation-induced oral mucositis (RIOM) is a common and debilitating complication of radiotherapy for head and neck cancer. Although Wumei extract (WME, *Prunus mume*) has been reported to have anti-inflammatory and mucosal protective effects, its therapeutic potential and underlying mechanisms in RIOM remain unclear.

**Methods:**

A mouse model of radiation-induced tongue dorsal mucositis (RITDM) was established using fractionated low-dose irradiation followed by a terminal high dose. Mice were treated with saline or WME, and outcomes including body weight, ulcer severity, histopathology, proliferation, apoptosis, and inflammatory cytokine expression were evaluated. Oral microbiota changes were analyzed using 16S rRNA and ITS sequencing. *In vitro*, irradiated human oral keratinocytes (HOK) were assessed for proliferation and migration with or without WME treatment.

**Results:**

WME significantly attenuated radiation-induced body weight loss, reduced ulcer severity, and preserved epithelial structure. It enhanced epithelial proliferation, reduced apoptosis, and downregulated TNF-α and IL-1β expression. Radiation enriched pro-inflammatory bacteria (*Rodentibacter pneumotropicus, Mammaliicoccus sciuri, Streptococcus acidominimus*) and fungi (*Fusarium oxysporum*), while WME treatment partially affected these dysbiotic profiles. *In vitro*, WME promoted proliferation and migration of irradiated HOK.

**Conclusion:**

WME alleviates RIOM by supporting epithelial regeneration, suppressing apoptosis and inflammation, and promotes oral microbiota balance, suggesting its potential as a novel adjunctive therapy for patients undergoing head and neck radiotherapy.

## 1 Introduction

Radiation-induced oral mucositis (RIOM) is a frequent and debilitating complication experienced by patients undergoing radiotherapy for head and neck cancers (Wakamori et al., 2022). Characterized by painful ulcerations, epithelial breakdown, and pronounced inflammation of the oral mucosa, RIOM severely impairs patients’ nutritional intake, oral hygiene, and overall quality of life, often resulting in treatment interruptions, increased healthcare costs, and a heightened risk of systemic infection (Zheng et al., 2020). Epidemiological studies indicate that up to 80% of head and neck radiotherapy patients develop some degree of oral mucositis, with approximately 65% experiencing severe lesions (Elting et al., 2007). Despite the widespread prevalence and clinical significance of RIOM, current therapeutic strategies remain largely supportive, with limited efficacy and frequent adverse effects. Standard interventions, such as topical anesthetics, anti-inflammatory agents, and antimicrobial rinses, provide only modest symptomatic relief and do not adequately address the underlying pathophysiological processes driving mucosal injury and delayed healing (Maria et al., 2017). This pressing clinical challenge highlights the urgent need for safe, effective, and mechanism-based therapies capable of both preventing and alleviating RIOM (Hong et al., 2019; Shuai et al., 2020).

The pathogenesis of RIOM is multifactorial, involving a series of interconnected pathological events. Radiation exposure initiates cellular and molecular damage, triggering inflammatory signaling pathways (Sonis, 2004; Lee and Galloway, 2022). This, in turn, activates the innate immune response, characterized by the release of proinflammatory cytokines, such as tumor necrosis factor-alpha (TNF-α) and interleukin-1β (IL-1β), and the recruitment of immune cells to the mucosal tissue. The resulting inflammation exacerbates epithelial injury and impairs mucosal repair, ultimately leading to ulceration and delayed healing (Chen C. et al., 2020; Lee and Galloway, 2022). This sequence is further compounded by the development of microbial dysbiosis within the oral cavity.

The oral microbiome is integral to mucosal health, serving as a protective barrier and modulator of immune responses. A balanced microbial community helps regulate inflammation and prevents colonization by pathogenic organisms (Nassar et al., 2017; Shang et al., 2018; Wilharm et al., 2019). However, radiation can profoundly disrupt this equilibrium, promoting the overgrowth of pro-inflammatory and pathogenic species (Hu Y. J. et al., 2013; Hu Y. et al., 2013). Such dysbiosis aggravates mucosal inflammation, enhances cytokine-mediated tissue injury, and impairs healing. Furthermore, radiation-induced epithelial damage increases susceptibility to microbial colonization, fueling a vicious cycle of inflammation and secondary infection that complicates the resolution of RIOM (Sonis, 2009; Zhu et al., 2017). Thus, interventions aimed at restoring oral microbial balance and suppressing excessive inflammatory responses may offer significant therapeutic benefits.

Traditional Chinese medicine (TCM) offers a rich source of natural compounds with anti-inflammatory, antimicrobial, and tissue-protective properties. Wumei extract (WME) has long been used in TCM for the management of gastrointestinal disorders (Liu et al., 2023; Deng et al., 2024; Liang et al., 2025). In traditional Chinese medicine, Wumei is regarded as a food-medicine resource because it has long been used both as an edible material and as a medicinal agent. Notably, WME has been shown to reduce mucosal inflammation, regulate gut microbial homeostasis, and promote tissue regeneration, suggesting potential applicability in the oral environment, where similar anatomical and physiological principles apply (Liu et al., 2023; Deng et al., 2024; Liang et al., 2025; Qian et al., 2025). However, the therapeutic potential and underlying mechanisms of WME in the context of RIOM remain poorly understood.

In this study, we aimed to comprehensively evaluate the therapeutic potential of WME in alleviating RIOM using a mouse model of radiation-induced tongue dorsal mucositis (RITDM). Through detailed analyses of epithelial cell proliferation and apoptosis, inflammatory cytokine expression, and oral microbial diversity, complemented by *in vitro* assessments of human oral keratinocyte (HOK) proliferation and migration, we sought to elucidate the multifaceted mechanisms by which WME may alleviate RIOM. Our findings not only provide novel insights into the therapeutic actions of WME but also offer a scientific rationale for its clinical application as an adjunctive treatment in patients undergoing radiotherapy for head and neck cancers.

## 2 Materials and methods

### 2.1 Preparation of WME for *in vivo* studies

Commercial decoction-free granules of WME were purchased from the Pharmacy of West China Hospital, Sichuan University (Chengdu, China), and were supplied by Sichuan New Green Pharmaceutical Technology Development Co., Ltd. (Chengdu, China). According to the supplier’s product information, the granules were prepared from the dried, nearly ripe fruit of the Rosaceae plant *Prunus mume* through water extraction, separation, concentration, drying, and granulation. The same batch of granules was used throughout the *in vivo* and *in vitro* experiments. WME suspension was prepared as follows: 432 g of WME granules were mixed thoroughly with 150 mL ultrapure water and dissolved by heating in a water bath. The resulting suspension (2.88 g/mL) was dispensed into 50 mL centrifuge tubes, sealed tightly, sterilized by ultraviolet irradiation, and stored at 4 °C until use.

### 2.2 Animal model and experimental grouping

Male BALB/c mice (7 weeks old, ∼20 g) were utilized to establish the RITDM model. For model optimization, 30 mice were randomly assigned to five groups (*n* = 6 per group): a non-irradiated control and four irradiation dose groups (3 Gy/day for 5 days, two cycles with a 2-day rest, followed by a single dose of 12, 14, 16, or 18 Gy) (Takashi et al., 2014; Maria et al., 2016; Tao et al., 2019; Chen J. et al., 2020). This pilot experiment was performed to determine the optimal irradiation dose for mucositis induction. For the intervention study, 18 mice were randomized into three groups (*n* = 6 per group): control (no irradiation), IRT + Saline (irradiation + saline), and IRT + WME (irradiation + Wumei extract). The control group did not receive irradiation and received once-daily swabbing of the oral mucosa with 1 mL saline (0.9%). The IRT + Saline group received irradiation and once-daily swabbing of the oral mucosa with 1 mL saline (0.9%). The IRT + WME group received irradiation and once-daily swabbing of the oral mucosa with 1 mL of WME (2.88 g/mL). In the irradiated groups, saline or WME treatment started three days before irradiation and continued throughout the irradiation period; control mice were treated with saline on the same schedule without irradiation. All animals were housed in a specific-pathogen-free (SPF) environment in the State Key Laboratory of Oral Diseases.

### 2.3 Radiation protocol

Mice received localized head and neck irradiation using a biological X-ray irradiator (Rad Source RS2000, United States) (Jonsson and Eriksson, 2015). The protocol consisted of fractionated low-dose X-ray exposure (3 Gy per day for five consecutive days), followed by a 2-day rest, repeated for two cycles (total 30 Gy), and then a single terminal high dose (12, 14, 16, or 18 Gy) as appropriate for each group (Takashi et al., 2014; Maria et al., 2016; Tao et al., 2019). During irradiation, mice were placed in custom-made restrainers, exposing only the head and neck, with the rest of the body shielded by a lead plate (Chang et al., 2014). The X-ray source operated at 160 kV and 25 mA with a 0.3 mm copper filter, delivering a dose rate of ∼1.14 Gy/min. No anesthesia was used to avoid confounding health effects and enhance model reproducibility. Mice were monitored daily for body weight and general health, including activity and oral lesion progression. In our model optimization experiment, particular attention was paid to adverse signs such as progressive ulcer formation and reduced activity. The 14 Gy terminal dose, providing a balance between lesion severity and animal viability, was selected for subsequent therapeutic evaluation. Mice were monitored daily for body weight, visible oral lesion progression, food intake, and activity as general indicators of health status. The effects of RIOM and the therapeutic effects of WME were further evaluated by methylene blue staining, H&E staining, and immunohistochemical analyses at the endpoint of the experiment.

### 2.4 Sample collection and methylene blue staining

Mice were humanely euthanized by CO_2_ inhalation (30%–70% chamber volume per minute) on day 8 after the final irradiation (experimental day 23). The entire tongue was excised and rinsed in phosphate-buffered saline (PBS). For ulcer assessment, tongues were first swabbed with 1% acetic acid, then stained with 1% methylene blue for 1 min. Excess dye was removed using 1% acetic acid, leaving only ulcerated regions stained. Images were captured and ulcer areas quantified using ImageJ software (National Institutes of Health, United States). Ulcer extent was expressed as a percentage of the dorsal tongue surface area.

### 2.5 Histological processing and H&E staining

Tongue tissues were fixed in 4% paraformaldehyde at 4 °C for 24 h, dehydrated in graded ethanol, cleared in xylene, and embedded in paraffin. Sections (5 μm) were cut and mounted on glass slides. Standard hematoxylin and eosin (H&E) staining was performed, and sections were evaluated for epithelial thickness and basal cell density using light microscopy. Measurements were taken from three random points in each section and averaged across six mice per group.

### 2.6 Immunohistochemistry (IHC) and terminal deoxynucleotidyl transferase dUTP nick end labeling (TUNEL) assays

Consecutive tongue sections were used for IHC staining of proliferating cell nuclear antigen (PCNA), tumor necrosis factor-α (TNF-α), and interleukin-1β (IL-1β), as well as TUNEL assays for apoptosis detection. After deparaffinization and rehydration, endogenous peroxidase was blocked, and antigen retrieval performed with 0.1% trypsin at 37 °C. Slides were blocked with 5% bovine serum albumin (BSA), incubated overnight at 4 °C with primary antibodies (PCNA, Abcam; TNF-α, Santa Cruz; IL-1β, Thermo Fisher) at recommended dilutions, or with TUNEL reaction solution (Beyotime, China). The following day, slides were incubated with horseradish peroxidase (HRP)-conjugated secondary antibodies and a streptavidin-biotin complex, then developed with 3,3′-diaminobenzidine (DAB) substrate. Sections were counterstained with hematoxylin, dehydrated, cleared, and mounted. Negative controls (omission of primary antibody or TUNEL reagent) confirmed specificity. For each animal, six non-overlapping fields were quantified by blinded investigators using ImageJ.

### 2.7 Oral microbiota sampling and sequencing

Oral mucosal swab samples were collected from all mice immediately prior to euthanasia. Sterile swabs were rubbed along the buccal and tongue mucosa, then stored at −80 °C. DNA was extracted, and the near-full-length bacterial 16S rRNA gene was amplified using primers 27F/1492R, while the fungal internal transcribed spacer (ITS) region was amplified using primers ITS1F/ITS4R (Supplementary Table 1). Sequencing was performed on a PacBio Sequel III platform. Raw data were processed to generate amplicon sequence variants (ASVs). Taxonomic annotation was performed using the RDP classifier (bacteria) and UNITE v8 (fungi). Microbial diversity, composition, and differential abundance analyses were performed on the Majorbio Cloud Platform (Majorbio, China). Alpha diversity metrics (Shannon, Simpson) and taxonomic barplots were generated. Kruskal–Wallis tests were used for group comparisons (*P* < 0.05 considered significant). All raw 16S rRNA and ITS amplicon sequencing data generated in this study have been deposited in the NCBI Sequence Read Archive (SRA) under BioProject accession number PRJNA1289768.

### 2.8 Cell culture and WME preparation for *in vitro* studies

Human oral keratinocytes (HOK; ATCC, United States) were cultured in Defined Keratinocyte-SFM (KSFM, Thermo Fisher) supplemented with growth factors, or Dulbecco’s modified Eagle’s medium (DMEM, Thermo Fisher, United States) with 10% fetal bovine serum (FBS, Thermo Fisher, United States), at 37 °C in a humidified 5% CO_2_ incubator. Cells were passaged at 80%–90% confluence and cryopreserved as required. WME for *in vitro* use was prepared from the same batch of instant granules, dissolved in DMSO, filtered for sterility, and diluted into culture medium to achieve final concentrations of 25, 50, 100, or 200 μg/mL.

### 2.9 CCK-8 cell viability and proliferation assays

Cell viability was evaluated using the Cell Counting Kit-8 (CCK-8, Solarbio, Beijing). For dose-response assays, HOK cells were seeded in 96-well plates (5 × 10^3^/well), incubated for 24 h, and treated with WME at indicated concentrations (25, 50, 100, 200 μg/mL) or vehicle control for 24 h. CCK-8 reagent was added, incubated for 1 h, and absorbance measured at 450 nm. For time-course proliferation, HOKs were treated with 200 μg/mL WME, and viability was assessed at 24, 48, and 72 h. Viability was calculated as a percentage relative to control.

### 2.10 Scratch wound healing assay

Human oral keratinocytes were seeded in 6-well plates and grown to confluence. Four groups were assessed: control, WME only, IRT (5 Gy), and IRT + WME (WME pretreatment + irradiation). WME-treated groups were pre-incubated with 200 μg/mL WME for 2 h before irradiation. A linear scratch was made with a pipette tip, wells rinsed, and cultured in serum-free medium. Images were captured at 0, 24, and 48 h. Wound closure was quantified using ImageJ, with results expressed as percent closure relative to the initial wound area.

### 2.11 Statistical analysis

Data are presented as mean ± standard deviation (SD). Statistical analyses were performed using GraphPad Prism 8.0. Group comparisons were assessed by one-way ANOVA with Tukey’s *post-hoc* test or, for two-group comparisons, unpaired two-tailed Student’s *t*-test. Microbiome analyses used Kruskal–Wallis rank-sum tests. Statistical significance was set at *P* < 0.05. All experiments were repeated at least in triplicate, with sample sizes described above.

## 3 Results

### 3.1 Fractionated irradiation successfully induced a stable RITDM mouse model

**FIGURE 1:**
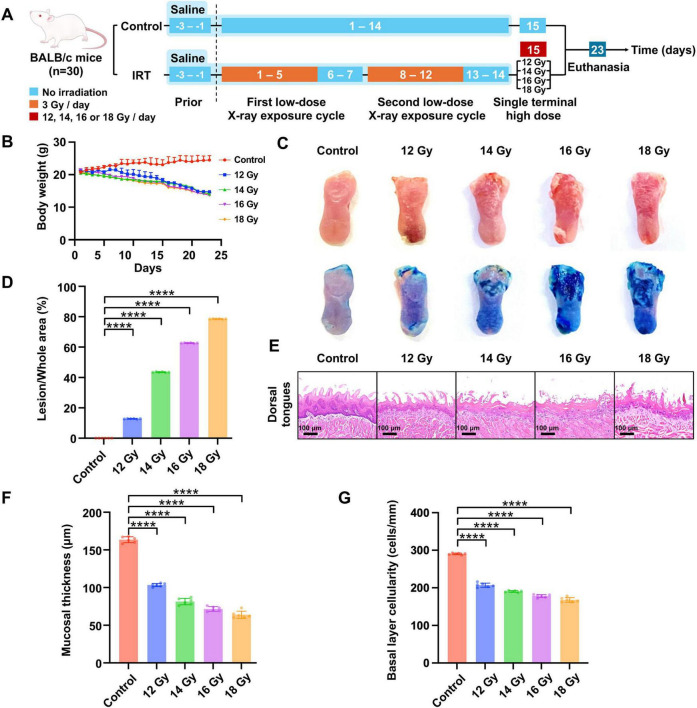
Establishment and characterization of a radiation-induced tongue dorsal mucositis (RITDM) mouse model using fractionated irradiation. **(A)** Experimental scheme for fractionated irradiation-induced RITDM model establishment, followed by different terminal high-dose exposures (12, 14, 16, or 18 Gy). **(B)** Body weight changes throughout the experimental period. **(C)** Representative tongue dorsal images before and after methylene blue staining. **(D)** Quantification of the methylene blue-positive lesion area as a percentage of the total tongue dorsal surface. **(E)** Representative H&E staining of tongue dorsal sections (scale bar = 100 μm). **(F,G)** Quantification of mucosal epithelial thickness **(F)** and basal layer cellularity **(G)**. (*n* = 6; ns; ****P < 0.0001).

To establish a stable and clinically relevant mouse model of RITDM, periodic low-dose (3 Gy) fractionated irradiation was employed to sensitize the oral mucosa, followed by a single high-dose irradiation (12, 14, 16, or 18 Gy) to induce mucositis on the dorsal surface of the tongue (Figure 1A). Throughout the experiment, the body weight of mice was closely monitored (Figure 1B). Following the terminal high-dose irradiation, body weight loss became more pronounced. Small oral ulcers were gradually observed at 5–6 days, and these lesions progressively coalesced into larger ulcers by days 7–8. Consistent with the progression of mucositis, mice showed reduced food intake and decreased activity during this period. Compared with the control group, all irradiated mice exhibited a gradual decrease in body weight; however, no statistically significant differences were observed among the irradiated groups. Methylene blue staining demonstrated the formation of tongue dorsal lesions in all irradiated groups (Figure 1C). Quantitative analysis of the methylene blue-positive lesion/whole area ratio showed a dose-dependent increase from 12.82%, 43.58%, 62.69%, and 78.54% in the 12, 14, 16, and 18 Gy groups, respectively, compared with 0% in the control group (*P* < 0.0001; Figure 1D). Histopathological analysis of the tongue dorsal epithelium via hematoxylin and eosin (H&E) staining demonstrated significant structural alterations in irradiated mice compared with controls. Compared with the intact and well-structured epithelium in the control group, irradiated mice displayed substantial disruption in mucosal continuity, pronounced inflammatory infiltration, reduced mucosal thickness, and decreased basal cell density (Figure 1E). Quantitative analyses further showed a significant reduction in mucosal thickness, decreased from 163.5 μm in the control group to 103.33, 81.50, 71.67, and 64.00 μm in the 12, 14, 16, and 18 Gy groups, respectively (*P* < 0.0001; Figure 1F), while basal layer cellularity decreased from 290.33 to 207.00, 190.33, 178.00, and 168.00 cells/mm, respectively (*P* < 0.0001; Figure 1G), indicating dose-dependent histopathological deterioration.

Together, selecting an appropriate terminal irradiation dose is critical to ensuring both the survival of experimental animals and the development of characteristic mucosal lesions. Based on the balance between disease severity and animal viability, 14 Gy was selected as the terminal radiation dose for subsequent therapeutic evaluation.

### 3.2 WME alleviates RITDM in mice

**FIGURE 2:**
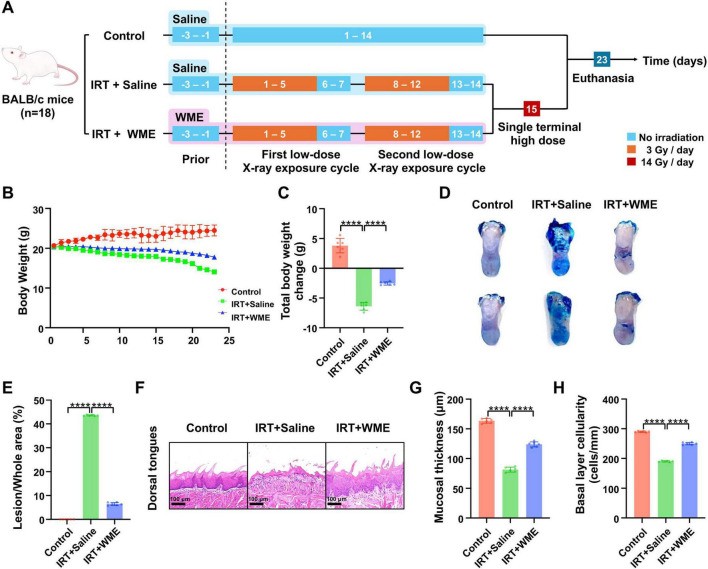
Wumei extract (WME) alleviates radiation-induced tongue dorsal mucositis (RITDM) in mice. **(A)** Experimental scheme for saline or WME treatment during fractionated irradiation in the RITDM mouse model. **(B)** Body weight changes throughout the experimental period. **(C)** Total body weight change at the end of the experiment. **(D)** Representative images of methylene blue-stained tongue dorsal mucosa. **(E)** Quantification of mucosal lesion as a percentage of the total dorsal surface. **(F)** Representative H&E-stained sections of tongue dorsal mucosa (scale bar = 100 μm). **(G,H)** Quantification of mucosal epithelial thickness **(G)** and basal layer cellularity **(H)**. (*n* = 6; ****P < 0.0001).

To evaluate the therapeutic potential of WME against RITDM, mice were subjected to fractionated irradiation with a terminal high-dose exposure of 14 Gy, as determined optimal in Figure 1. Mice receiving irradiation treatment (IRT) were treated daily with either saline solution (IRT+Saline group) or WME solution (IRT+WME group) from 3 days before irradiation until the end of fractionated low-dose irradiation (Figure 2A).

Throughout the experiment, body weights were continuously monitored (Figure 2B). Total body weight change was 3.81 g in the control group, −6.39 g in the IRT+Saline group, and −2.55 g in the IRT+WME group, indicating that WME significantly attenuated irradiation-induced weight loss compared with saline treatment (*P* < 0.0001; Figure 2C). Methylene blue staining revealed substantial differences in tongue dorsal mucosal lesion severity among groups (Figure 2D). The control group exhibited smooth, intact tongue mucosa with minimal staining, whereas the irradiated group receiving saline treatment showed extensive tongue dorsal lesions with strong methylene blue staining. In contrast, WME-treated irradiated mice displayed scattered, smaller mucosal lesions with noticeably reduced staining intensity. Quantitative analysis showed that the lesion/whole area ratio was 43.58% in the IRT+Saline group and 6.47% in the IRT+WME group, compared with the control group, demonstrating that WME significantly reduced lesion severity (*P* < 0.0001; Figure 2E).

Histopathological analysis via H&E staining further confirmed the protective effects of WME (Figure 2F). Compared with the control group and IRT+WME group, tongue dorsal mucosal sections from the IRT+Saline group showed severe epithelial disruption, marked inflammatory cell infiltration, decreased mucosal thickness, and diminished basal cell density (Figures 2G, H). Quantitative analysis showed that mucosal thickness decreased from 163.50 μm in the control group to 81.50 μm in the IRT+Saline group, while WME treatment increased it to 123.83 μm (*P* < 0.0001; Figure 2G). Similarly, basal layer cellularity decreased from 290.33 cells/mm in the control group to 190.33 cells/mm in the IRT+Saline group, and increased to 250.17 cells/mm in the IRT+WME group (*P* < 0.0001; Figure 2H). Together, these findings indicate that WME effectively alleviates RITDM in mice.

### 3.3 WME enhances epithelial proliferation, reduces apoptosis, and suppresses inflammation in the tongue dorsal mucosa of RITDM mice

**FIGURE 3:**
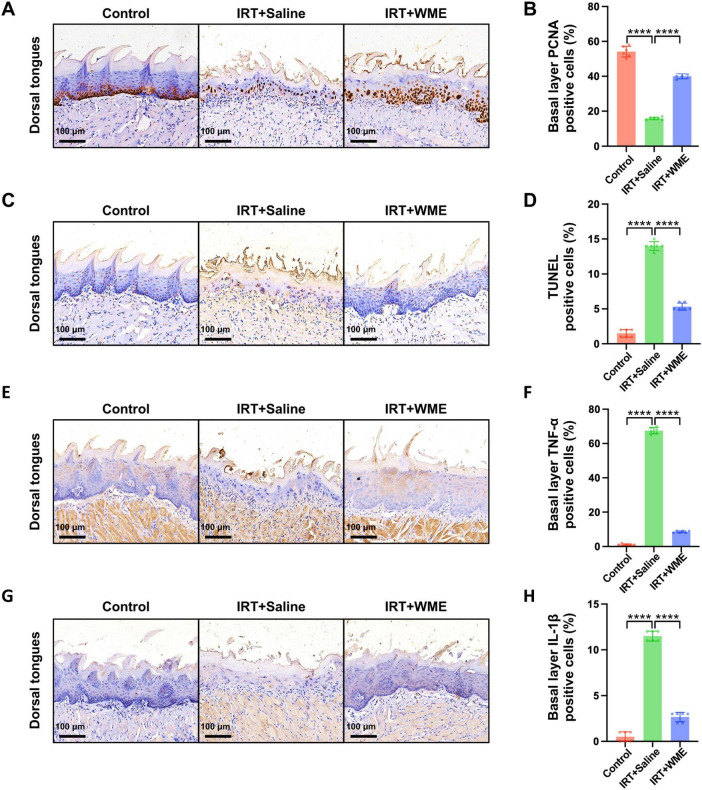
Wumei extract (WME) enhances epithelial proliferation, reduces apoptosis, and suppresses inflammation in the tongue dorsal mucosa of radiation-induced tongue dorsal mucositis (RITDM) mice. **(A,B)** Representative proliferating cell nuclear antigen (PCNA) staining **(A)** and quantification of PCNA-positive cells **(B)**. **(C,D)** Representative terminal deoxynucleotidyl transferase dUTP nick end labeling (TUNEL) staining **(C)** and quantification of TUNEL-positive cells **(D)**. **(E,F)** Representative tumor necrosis factor-alpha (TNF-α) staining **(E)** and quantification of TNF-α-positive cells **(F)**. **(G,H)** Representative interleukin-1β (IL-1β) staining **(G)** and quantification of IL-1β-positive cells **(H)**. (Scale bar = 100 μm; *n* = 6; ****P < 0.0001).

To elucidate the mechanisms underlying the protective effects of WME in RITDM, epithelial cell proliferation, apoptosis, and inflammatory cytokine expression were assessed in mouse tongue dorsal mucosa by immunohistochemistry. PCNA staining showed that the percentage of PCNA-positive basal cells was significantly reduced in the IRT+Saline group compared with the control group from 54.17% to 15.83% (*P* < 0.0001), whereas the percentage of PCNA-positive basal cells was significantly increased in IRT+WME group compared with IRT+Saline group from 15.83% to 40.00% (*P* < 0.0001; Figures 3A, B). TUNEL staining was used to assess epithelial apoptosis (Figures 3C, D). The percentage of TUNEL-positive cells increased from 1.50% in the control group to 14.00% in the IRT+Saline group (*P* < 0.0001), and was reduced in IRT+WME group compared with IRT+Saline group from 14.00% to 5.33% (*P* < 0.0001; Figure 3D), suggesting that WME protected against radiation-induced epithelial cell death.

To further assess the anti-inflammatory properties of WME, immunohistochemical staining for the classical proinflammatory cytokines TNF-α and IL-1β was performed (Figures 3E–H). The IRT+Saline group exhibited significantly increased expression levels of both TNF-α and IL-1β within the tongue dorsal epithelium compared with the control group (Figures 3E–H). TNF-α-positive cells were increased in the IRT+Saline group compared with the control group from 1.17% to 67.50% (*P* < 0.0001), and were reduced in the IRT+WME group compared with IRT+Saline group from 67.50% to 8.50% (*P* < 0.0001; Figure 3F). Similarly, IL-1β-positive cells increased in the IRT+Saline group compared with the control group from 0.50% to 11.50% (P < 0.0001), and decreased in the IRT+WME group compared with IRT+Saline group from 11.50% to 2.67% (*P* < 0.0001; Figure 3H). Together, these results indicate that WME alleviates RITDM by promoting epithelial cell proliferation and inhibiting apoptosis, and attenuating radiation-induced inflammatory responses in the tongue dorsal mucosa.

### 3.4 WME treatment changes the microbial composition in RITDM mice

**FIGURE 4:**
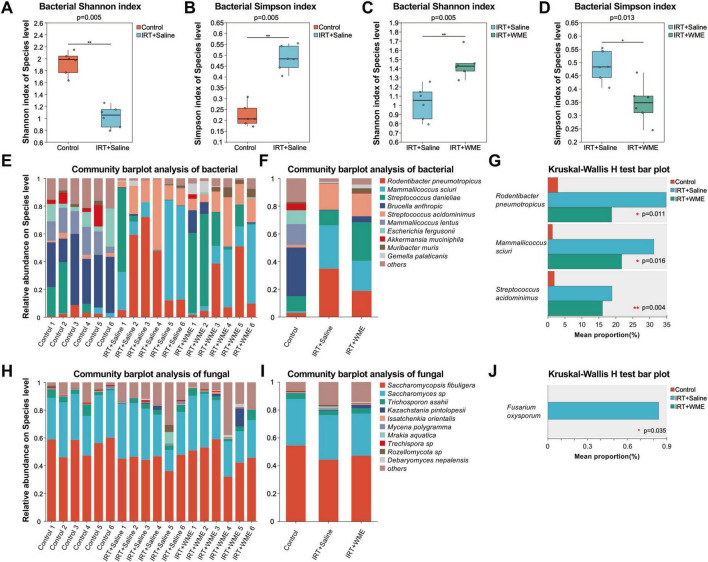
Wumei extract (WME) alleviates oral microbiota dysbiosis in radiation-induced tongue dorsal mucositis (RITDM) mice. **(A,B)** Bacterial alpha diversity in the control and IRT+Saline groups as measured by Shannon index **(A)** and Simpson index **(B)**. **(C,D)** Bacterial alpha diversity in the IRT+Saline and IRT+WME groups as measured by Shannon index **(C)** and Simpson index **(D)**. **(E)** Community bar plot analysis of bacterial species-level composition for each mouse. **(F)** Group average community bar plot analysis of bacterial species-level composition. **(G)** Differential abundance analysis of key bacterial species among groups. **(H)** Community bar plot analysis of fungal species-level composition for each mouse. **(I)** Group average community bar plot analysis of fungal species-level composition. **(J)** Differential abundance analysis of key fungal species among groups. (*n* = 6, *P < 0.05, **P < 0.01).

To characterize oral microbial changes associated with RITDM and WME treatment, microbial diversity and composition were analyzed among the control, IRT+Saline, and IRT+WME groups. Compared with the control group, the IRT+Saline group exhibited significant lower bacterial alpha diversity, with a reduced Shannon index (1.93 vs. 1.03, *P* = 0.005; Figure 4A and Supplementary Table 2) and an increased Simpson index (0.22 vs. 0.49, *P* = 0.005; Figure 4B and Supplementary Table 2). Compared with the IRT+Saline group, the IRT+WME group showed an increased Shannon index (1.03 vs. 1.44, *P* = 0.005; Figure 4C and Supplementary Table 3) and a reduced Simpson index (0.49 vs. 0.35, *P* = 0.013; Figure 4D and Supplementary Table 3). Community composition analysis at the species level also revealed irradiation-associated changes in bacterial structure. Specifically, the relative abundances of *Rodentibacter pneumotropicus*, *Mammaliicoccus sciuri*, and *Streptococcus acidominimus* were increased in the IRT+Saline group (*P* < 0.05; Figures 4E–G).

In addition to bacterial shifts, irradiation was also associated with changes in the oral fungal community. Compared with the control group, the IRT+Saline group exhibited significantly higher Shannon and lower Simpson indices, indicating increased fungal richness and evenness (Supplementary Tables 4,5). In the IRT+WME group, Shannon values tended to decrease and Simpson values tended to increase relative to the IRT+Saline group, although these differences did not reach statistical significance. The fungal community analysis similarly indicated radiation-induced dysbiosis, with a significant increase in the pathogenic fungus *Fusarium oxysporum* observed in the IRT+Saline group (*P* = 0.035; Figures 4H–J). WME treatment was associated with a reduced relative abundance of *F. oxysporum*, restoring the fungal community to a composition closer to that observed in the non-irradiated control group. Collectively, these results show that WME affects bacterial and fungal composition.

### 3.5 WME promotes proliferation and migration of radiation-induced HOK cells *in vitro*

**FIGURE 5:**
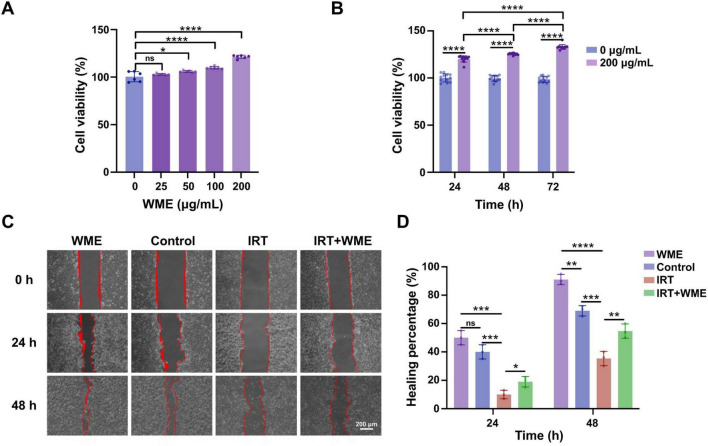
Wumei extract (WME) promotes proliferation and attenuates radiation-induced migration impairment in human oral keratinocytes (HOK) cells. **(A)** CCK-8 assay showing HOK cell viability after treatment with various concentrations of WME (0, 25, 50, 100, and 200 μg/mL) for 24 h (*n* = 6). **(B)** Time-dependent proliferation of HOK cells treated with 200 μg/mL WME measured at 24, 48, and 72 h (*n* = 12). **(C)** Representative wound-healing images at 0, 24, and 48 h. (Scale bar = 200 μm). **(D)** Quantification of wound healing percentages at 24 and 48 h (*n* = 3). (ns, not significant, *P < 0.05, **P < 0.01, ***P < 0.001, ****P < 0.0001).

To evaluate the bioactivity of WME on HOK cells, we first assessed its effect on cell proliferation using the CCK-8 assay. HOK cells were treated with varying concentrations of WME (0, 25, 50, 100, 200 μg/mL) for 24 h. WME increased HOK cell viability in a concentration-dependent manner, with viability rising from 100.48% in untreated cells to 106.03%, 109.99%, and 121.27% after treatment with 50, 100, and 200 μg/mL WME, respectively (*P* < 0.0001; Figure 5A). Based on these findings, a concentration of 200 μg/mL was selected for subsequent experiments. To determine the time-dependent effect of WME on HOK proliferation, cells were treated with 200 μg/mL WME and assessed at 24, 48, and 72 h. Compared with untreated cells, WME significantly increased HOK cell viability from 100.17%, 99.71%, and 98.67% to 120.23%, 125.56%, and 133.00% at 24, 48, and 72 h, respectively (*P* < 0.0001; Figure 5B). The most pronounced increase was observed at 72 h.

In addition, wound-healing assays were performed to evaluate the effect of WME on irradiated and unirradiated HOK. Confluent HOK monolayers were mechanically scratched across four experimental groups: control (no radiation or WME), WME (200 μg/mL WME only), IRT (5 Gy irradiation only), and IRT+WME (200 μg/mL WME pretreatment followed by 5 Gy irradiation), and images were captured at 0, 24, and 48 h to assess cell migration ability into the wound area (Figure 5C). The healing percentage was significantly decreased in the IRT group compared with the control group from 40% to 10% in 24 h (*P* = 0.0009; Figure 5D), 69% to 35% in 48 h (*P* = 0.0007; Figure 5D), whereas it increased in the IRT+WME group compared with the IRT group from 10% to 19% in 24 h (*P* = 0.0293; Figure 5D), 35% to 54% in 48 h (*P* = 0.0093; Figure 5D). At this time point, wound closure remained significantly impaired in the IRT group compared with the control group, while the IRT+WME group showed partial restoration of migratory capacity relative to the IRT group. The healing percentage was significantly increased in the WME group compared with the control group from 69% to 91% in 48 h (*P* = 0.0017; Figure 5D). Together, these results suggest that WME promotes HOK cell proliferation and migration, and significantly protects against radiation-induced migration impairment.

## 4 Discussion

In this study, WME showed protective effects against RIOM in a mouse model, with cytoprotective effects on HOK *in vitro*. Our findings show that WME alleviates mucosal damage, reduces inflammation, promotes epithelial regeneration, and changes oral microbiota composition. Collectively, these findings suggest that the protective effects of WME may involve combined anti-inflammatory, epithelial-protective, and alterations of oral microbiota.

Consistent with previous reports of mucosal injury after irradiation, our RITDM mouse model exhibited pronounced weight loss and epithelial disruption (Han et al., 2013; Yang et al., 2018). WME intervention reduced weight loss, preserved mucosal structure, and improved both mucosal thickness and basal cell density, suggesting that WME supports epithelial renewal and integrity during radiation exposure. Immunohistochemical analyses further confirm that WME enhanced basal epithelial proliferation (as indicated by PCNA staining) while simultaneously reducing apoptosis (as shown by TUNEL assays). These findings indicate that WME enhanced epithelial regeneration and reduced cell loss. WME also reduced the expression of pro-inflammatory cytokines, including TNF-α and IL-1β, in the tongue mucosa. As these cytokines are major mediators of tissue damage and delayed healing in RIOM, their downregulation by WME may alleviate oral mucositis primarily by suppressing inflammatory injury and promoting epithelial repair (Yang et al., 2018; Pulito et al., 2020).

The oral microbiota results further showed that WME treatment changed radiation-induced oral microbial dysbiosis. Our results showed that irradiation disrupted the bacterial and fungal community structure, enriching potentially opportunistic taxa like *R. pneumotropicus*, *M. sciuri*, *S. acidominimus*, and *F. oxysporum*, which are associated with mucosal inflammation and delayed wound healing (Sasaki et al., 2013; Wu et al., 2014; Kwon et al., 2023; Samborska et al., 2024; Ayhan et al., 2025). WME treatment was associated with lower relative abundances of these taxa and with microbial community profiles that were closer to those of non-irradiated controls. Relative to the control group, the IRT+Saline group showed a dysbiotic irradiated profile, whereas the IRT+WME group displayed a partial shift toward a community structure more similar to that of the non-irradiated controls, particularly at the bacterial level. These findings are broadly consistent with previous studies showing that oral microbial alterations may contribute to mucosal injury and delayed healing in oral mucositis (Vasconcelos et al., 2016; Min et al., 2023).

Our *in vitro* findings using HOK provide further support for the regenerative potential of WME. WME promoted HOK proliferation in a dose- and time-dependent manner and significantly improved wound closure, even in the context of irradiation-induced migration impairment. These results indicate that WME supports the recovery of oral epithelial cells after radiation damage, highlighting its utility for both prophylactic and therapeutic application.

Radiation-induced oral mucositis is a prevalent and debilitating complication for patients receiving radiotherapy for head and neck cancers, often resulting in treatment delays, decreased quality of life, and increased risk of infection (Hong et al., 2019; Xia et al., 2025). The multifaceted actions of WME, including anti-inflammatory, epithelial-protective effects, and microbial changes, suggest that it may serve as an effective adjunctive therapy for preventing or alleviating mucositis in this patient population.

Although the precise bioactive compounds of WME were not identified in this study, previous studies have suggested that Wumei-related preparations may exert anti-inflammatory, microbiota-regulating, and tissue-protective effects in mucosal disease settings (Liu et al., 2023; Deng et al., 2024; Liang et al., 2025; Qian et al., 2025). Wumei-related preparations have been reported to increase short-chain fatty acids and suppress TLR4/MyD88/NF-κB signaling in intestinal mucositis, while Mume Fructus-induced microbial changes were associated with host mucosal barrier markers such as ZO-1, Occludin, Claudin-1, and Muc2 (Lu et al., 2022; Yang et al., 2024). *In vitro* evidence also showed that Wumei extract directly inhibited multiple oral bacterial species, including several *Streptococcus* species (Seneviratne et al., 2011).

In summary, our study demonstrates that WME alleviates radiation-induced oral mucositis by enhancing epithelial regeneration, suppressing apoptosis and inflammation, and change in oral microbial composition. These findings establish a scientific rationale for further clinical investigation of WME as a novel therapeutic option for patients undergoing radiotherapy for head and neck cancers.

Despite these promising findings, several limitations must be acknowledged. The mouse model, while informative, does not fully recapitulate the complexity of human RIOM, and further clinical studies are needed to validate the efficacy and safety of WME in patients. Additionally, although we observed significant microbiota changes, deeper analyses are necessary to identify key microbial players and clarify the causal relationships between microbiota modulation and mucosal healing. The precise bioactive compounds in WME responsible for the observed effects were not identified, and further phytochemical characterization is needed.

## Data Availability

The raw 16S rRNA and ITS amplicon sequencing data are deposited in the NCBI Sequence Read Archive under BioProject accession number PRJNA1289768.
