# Supplementary material for: Wumei extract modulates inflammation and oral microbiota for radiation-induced oral mucositis
Source: Front Microbiol. 2026 Aug 4;17:1837933. doi: 10.3389/fmicb.2026.1837933 (PMC13481452; doi:10.3389/fmicb.2026.1837933)
Supplement: Supplementary file 1 [file Data_Sheet_1.docx]

**Supplementary Materials**

**Table S1. Primers used for amplification of bacterial 16S rRNA and fungal ITS regions.**

| **Target** | **Primer Name** | **Primer Sequence (5’-3’)** |
| --- | --- | --- |
| 27F_1492R (Bacteria) | 27F | AGRGTTYGATYMTGGCTCAG |
|  | 1492R | RGYTACCTTGTTACGACTT |
| ITS1F_ITS4R (Fungi) | ITS1F | CTTGGTCATTTAGAGGAAGTAA |
|  | ITS4R | TCCTCCGCTTATTGATATGC |

**Table S2.** **Alpha diversity of bacterial communities in the control and IRT+Saline groups.**

| **Estimators** | **Control Mean** | **Control SD** | **IRT+Saline Mean** | **IRT+Saline SD** | ***P* value** |
| --- | --- | --- | --- | --- | --- |
| **Shannon** | 1.925 | 0.191 | 1.026 | 0.178 | 0.005 |
| **Simpson** | 0.222 | 0.051 | 0.485 | 0.057 | 0.005 |

**Table S3.** **Alpha diversity of bacterial communities in the IRT+Saline and IRT+WME groups.**

| **Estimators** | **IRT+Saline Mean** | **IRT+Saline SD** | **IRT+WM Mean** | **IRT+WME SD** | ***P* value** |
| --- | --- | --- | --- | --- | --- |
| **Shannon** | 1.026 | 0.178 | 1.441 | 0.138 | 0.005 |
| **Simpson** | 0.485 | 0.057 | 0.347 | 0.073 | 0.013 |

**Table S4. Alpha diversity of fungal communities in the Control and IRT+Saline groups.**

| **Estimators** | **Control Mean** | **Control SD** | **IRT+Saline Mean** | **IRT+Saline SD** | ***P* value** |
| --- | --- | --- | --- | --- | --- |
| **Shannon** | 1.190 | 0.200 | 1.703 | 0.463 | 0.020 |
| **Simpson** | 0.416 | 0.058 | 0.312 | 0.072 | 0.020 |

**Table S5. Alpha diversity of fungal communities in the IRT+Saline and IRT+WME groups.**

| **Estimators** | **IRT+Saline Mean** | **IRT+Saline SD** | **IRT+WM Mean** | **IRT+WME SD** | ***P* value** |
| --- | --- | --- | --- | --- | --- |
| **Shannon** | 1.703 | 0.463 | 1.407 | 0.305 | 0.230 |
| **Simpson** | 0.312 | 0.072 | 0.353 | 0.082 | 0.689 |
